# Smokeless Tobacco Products (STPs) Harbour Bacterial Populations with Potential for Oral Carcinogenicity

**DOI:** 10.31557/APJCP.2020.21.3.815

**Published:** 2020-03

**Authors:** Srivastav Monika, Thayalan Dineshkumar, Shankaran Priyadharini, Thampan Niveditha, Priyadharshini SK, Krishnan Rajkumar

**Affiliations:** *Department of Oral Pathology and Microbiology, SRM Dental College, Ramapuram, SRM Institute of Science and Technology, Chennai, Tamil Nadu, India. *

**Keywords:** Bacterial population, smokeless Tobacco products, oral carcinogenicity, 16S rRNA

## Abstract

**Introduction:**

Smokeless Tobacco Products (STPs) vary significantly in their carcinogenicity, a feature accredited to the variation in the concentrations of carcinogenic chemicals. Tobacco associated bacteria are known to produce Tobacco-specific N-nitrosamines (TSNAs) and hence are determinants of TSNA levels in Tobacco. The primary objective of this study was to conduct a microbiological survey of STPs and to provide a baseline information of the bacterial communities present in the STPs.

**Materials and Methods:**

The present study analyzed the constituency of microbial communities in 7 different smokeless Tobacco products including four chewable (T1_CW to T4_CW), two snus (T5_Snus and T6_Snus) and one snuff sample (T7_Snuff) using high-throughput sequencing of the 16S rRNA based next generation sequencing. The Tobacco samples were also analyzed for pH and moisture content. Statistical analysis of the data obtained was done using SPSS software version 20. Pearson’s Correlation was done to analyze the correlation between pH and moisture content of the Tobacco samples.

**Results:**

A total of 11 phyla were identified in all smokeless Tobacco products. A total of 36 classes were identified across all smokeless Tobacco products and bacilli was the predominant class in all the products followed by Actinobacteria and Bacteroidia. In species level, a total of 2369 species were identified across all smokeless Tobacco products. In T1 chewable Tobacco products, predominant species was staphylococcus whereas in T2 and T3, Bacillus subtilis and pumilus were predominant. In T4 chewable Tobacco product, Virgibacillus was predominant followed by halodentrificans, staphylococcus epidermidis. In snus 1 and 2, Bacillus pumilus and subtilis were predominant. In snuff, Bacillus cereus was predominant. Snus products had the highest moisture content (15.4% and 14.3%) compared to the chewable Tobacco and snuff products. The snus products analyzed had alkaline values (pH 8.50 and 8.15) and snuff and chewable Tobacco had acidic values ranging from 5.62 to 6.09.

**Conclusion:**

The current study demonstrates that ST products differ qualitatively, quantitatively, and in their bacterial composition. There is a possibility that some of these species may contribute to oral carcinogenesis, either by influencing levels of TSNAs or directly inducing chronic inflammation.

## Introduction

Tobacco is the only officially permitted drug that is conscientious for killing many of its users when used exactly as intended by its manufacturers. WHO has gauged that Tobacco use currently accounts for the mortality of about six million people across the planet every year with many of these occurring prematurely (Bilano et al., 2005). The Global Adult Tobacco Survey (GATS) in India has estimated that 99.5 million (10.7%) of adults currently smoke Tobacco and 199.4 million (21.4%) of adults use the smokeless form of Tobacco. It is also predicted that there are more than 300 million users of smokeless Tobacco globally (Gupta and Ray, 2003; Bhawna, 2013).

Cigarette smoking is pandemic, whereas consumption of smokeless Tobacco can best be described as endemic, restricted to certain populations or countries. The use of smokeless Tobacco products is more common in North America, some of the Scandinavian countries, Saudi Arabia, India, Bangladesh, Southeast Asia, and some parts of Africa (Asplund, 2014). 

As the name indicates, smokeless Tobacco products (STPs) are consumed without smoking (heating), and some consumers use it as an alternative to smoking cigarettes as there is a misconception that STPs are less harmful, even though there is substantial evidence to support carcinogenicity of STPs. According to the “International Agency for Research on Cancer” (IARC), which assesses the carcinogenic risk, there is sufficient evidence to advocate carcinogenicity of STPs in its users, so as to consider it as a cause of cancers of the oral cavity (Han et al., 2016; Al-hebshi et al., 2017). 

However, different STPs seem to considerably differ in respect to their potential carcinogenicity, a feature accredited to the variation in the concentrations of carcinogenic chemicals, primarily Tobacco-specific N-nitrosamines (TSNAs). The STPs marketed in Europe and America seem to have lesser concentrations of TSNAs and other chemical carcinogens compared to Indian products, which may partially explain the increased occurrence of oral cancers in Indian population due to STP use (Muwonge et al., 2008). 

Over the years, the detrimental health effects and carcinogenic effects of STPs have primarily focused only on their chemical composition and the presence of carcinogenic compounds (Borgerding et al., 2012). But like any other consumer product, there is a very high probability for microbial carriage in the Tobacco product that may be a health concern.

Despite believing that the microbial content plays an important role in accounting for the variation in the carcinogenicity of STPs, this notion has been understudied and unexplored. The Tobacco associated bacteria are known to produce nitrite by the reduction of nitrates, which in turn reacts with the alkaloids in Tobacco to form TSNAs and hence are determinants of TSNA levels in Tobacco (Wei et al., 2014; Ammann et al., 2015). Another probable risk associated with bacterial contamination is the production of metabolic by-products that may be harmful to the individuals consuming STPs (Al-hebshi et al., 2017). 

However, very little research has been performed on the bacterial communities present in smokeless Tobacco products and their expressed activities. The microbial content is greatly affected by the processing procedure of Tobacco, its manufacturing method and also the water content plays a role in increasing the microbial population as water content supports the microbial growth.

Previous studies on the microbiota present in Tobacco products conventionally utilized culture-based methods that could probably under represent the diversity of microbiome. However, there are only a few studies that have used 16S rDNA metagenomic analysis to analyze the Tobacco products, which will provide an accurate representation of the bacterial communities present.

But one of the limitation of these studies is that, most of these methods have utilized only the standard methods of interrogating the hypervariable V3-V4 regions alone of the bacterial genome, which could potentially give only 60% of bacterial coverage. Whereas, a panel covering all V1-V9 regions can provide sensitive detection and accurate representation of each species in the sample compared to standard methods interrogating the V3-V4 region alone.

With these above facts, the primary objective of this study was to conduct a microbiological survey of STPs and to provide a baseline information of the bacterial communities present, and to gain a better understanding and expand the present knowledge of the microbial population that is present in smokeless Tobacco products.

## Materials and Methods

*Tobacco Samples*

Seven smokeless Tobacco products (STPs) were purchased from retail location in North India. The STPs were categorized into chewable Tobacco, snus and snuff. These products were stored at room temperature and prior to analysis, the unopened packages were placed under Ultraviolet light in a biological safety cabinet for 30 min to prevent contamination from exterior microorganisms. 

*Measurement of Moisture content and pH of STPs*

Total moisture content (%) was determined by the weight difference of fresh and dried Tobacco by evaporation method by measuring the mass of Tobacco before and after the water content removal by evaporation. A 1.0 g of Tobacco sample was weighed using Uni-Bloc, Analytical Balance - ATX224 (Shimadzu, Japan) and the weighed sample was dried using Magnetic Hot Plate (DBK, India) for 30 min at 100°C and their dried mass was determined again using Uni-Bloc, Analytical Balance. 

The pH was determined for all seven samples by weighing 1.0 g of Tobacco and adding it with 10 ml of deionized distilled water. The samples were vortexed for 5 min, and the pH was measured using Cyberscan pH Tutor (EuTech, Thermo Fisher Scientific, USA).

*16S rRNA Metagenome Analysis*

*DNA extraction*

DNA was extracted from Tobacco samples using FavorPrep DNA Isolation Mini Kit (Favorgen, Taiwan) as per the manufacturer instructions. The extracted DNA was quantified using Qubit 4 Fluorometer (Life Technologies, USA).

*16S rRNA PCR*

A PCR targeting 16S rRNA gene was performed for the seven different Tobacco samples with 25µL reaction volume consisting of broad-range pan 16S rRNA primers; 16S Fp-5’- AGAGTTTGATCCTGGCTCAG-3’ and 16S Rp-5’ ACGGCTACCTTG TTACGACTT-3’ (10 pM of each primer) as described by Weisburg (1991). The PCR amplification was performed in Veriti 96-Well Thermal Cycler (Applied Biosystems, USA) with initial denaturation at 95°C for 3 min followed by 35 cycles at 95^o^C for 30 seconds, 55^o^C for 30 seconds and 72^o^C for 1 min, and with final extension at 72^o^C for 7 min. Known positive and negative controls were also included. After 16S rRNA PCR, the amplicons were resolved along with DNA markers in 0.8% agarose with ethidium bromide (10 mg/mL) by gel electrophoresis for ~25min at 135 V using Mupid-exU system (Takara, Japan) and gel was analysed by BioGlow UV Transilluminators (Crystal Technology, USA). The resultant product size is 1500bp.

*16S rRNA PCR Product Purification*

16S rRNA PCR amplicons were purified using FavorPrep PCR Purification Mini Kit (Favorgen, Taiwan) and after the purification amplicons was quantified using Qubit 4 Fluorometer for Nanopore library preparation.

*16S rRNA sequencing using Oxford Nanopore Technologies*

Sequencing was done using third-generation sequencing technology (TGS), Oxford Nanopore Technologies (ONT) - MinION Platform (Oxford Nanopore, UK). Briefly, 1 µg of 16S rRNA PCR product was used for the end repair process with NEBNext Ultra II End-repair/dA-tailing (New England Biolabs, USA). Then the end repaired DNA was purified using 1:1 Agencourt AMPure XP beads (Beckman Coulter Inc. USA), The recovery was evaluated with the Qubit 4.0 dsDNA BR Assay Kit. DNA Barcoding was done using 1D Native barcoding DNA Kit (with EXP-NBD104, EXP-NBD114, and SQK-LSK109) and Blunt/TA ligase MM (New England Biolabs, USA). The Barcoded PCR product was purified using 1:1 Agencourt AMPure XP beads (Beckman Coulter Inc. USA), Barcoding adapter were added to the ~700 ng barcoded ligated amplicons using native barcoding Adapter Mix II (AMII) with Quick T4 DNA Ligase (New England Biolabs, USA). The barcoding adapter PCR product was purified using 1:1 Agencourt AMPure XP beads (Beckman Coulter Inc. USA), Then 250 μl S Fragment Buffer (SFB) was added for the shorter reads size selection and the DNA library was pelleted onto magnet, finally, the sequencing DNA library was eluted in 15 µL of elution buffer (ELB) - pre-sequencing mix. To the sequencing DNA library mix Sequencing Buffer (SQB) and Library Loading beads (LLB) were added. Finally, the samples were loaded to the flow cell - FLO-MIN106 (Oxford Nanopore, UK) and sequencing was performed using MinION instrument (Oxford Nanopore, UK).

*16S rRNA Metagenome Data analysis*

The Fast5 output sequences from the MinION sequencer were basecalled and Demultiplexed using Albacore Software v2.0.1 and basecalled Fast5 sequences were converted to Fasta files using Poretools Software v0.5.1. The Adapters/barcodes were removed using Porechop Software v0.2.1.

*MG-RAST analysis*

The 16S rRNA processed reads were finally analysed using MG-RAST server - a metagenomics analysing server (http://www.mg-rast.org/) to understand the bacterial abundance and diversity.

## Results

*Moisture Content and pH of Tobacco samples*

**Figure 1:**
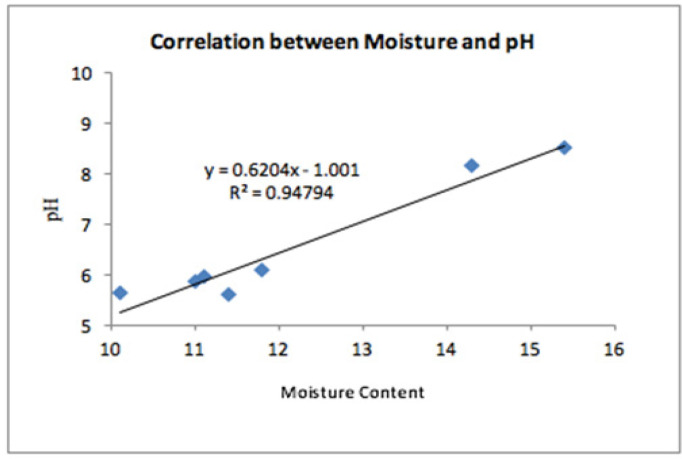
Correlation between Moisture Content and pH of Smokeless Tobacco Products

In terms of moisture content, snus products had the highest moisture content (15.4% and 14.3%) compared to the chewable Tobacco and snuff products. Percentage moisture in chewable Tobacco ranged between 10.1% and 11.8% and snuff had a value of 11.4%. In this current study, the STP products examined had pH ranging from 5.62 to 8.50. The snus products analyzed had alkaline values (pH 8.50 and 8.15) and snuff and chewable Tobacco had acidic values ranging from 5.62 to 6.09 (Table 1). On correlating the data of pH with moisture content, there was a positive correlation (Correlation coefficient r= 0.974, p value <0.001, Figure 1).

*Relative abundance of bacterial communities*

In terms of diversity of species, Alpha diversity is the measure of diversity in a single sample and T5_Snus showed high number of significant species (168 species) in the sample and T1_CW (chewable Tobacco) with the lowest number (33 species) of species diversity (Table 2).

**Figure 2:**
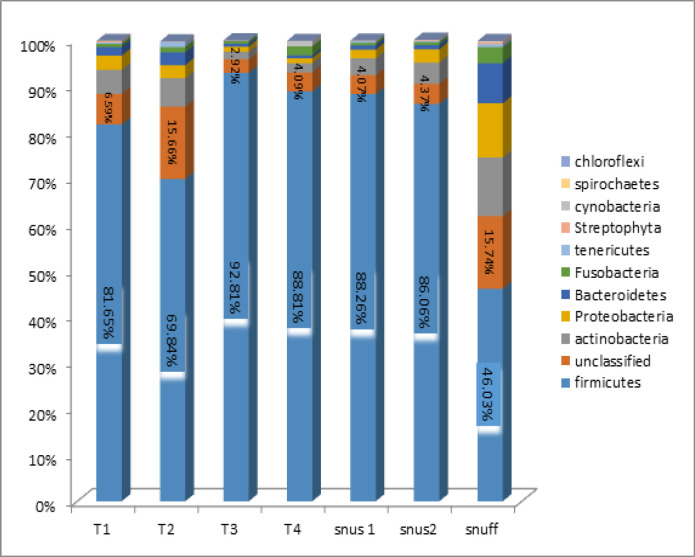
Bacteriome of Smokeless Tobacco Products - Phylum Level. (Stacked bars showing relative abundance of bacterial phyla identified in each of the ST product)

ST samples exhibited a wide range of taxonomic diversity. A total of 11 phyla were identified in the smokeless Tobacco products as shown in Figure 2. Phylum Firmicutes accounted for the majority of the sequences across all Tobacco products. The other phyla were Actinobacteria, Proteobacteria, Bacteroidetes, Fusobacteria, Tenericutes, Streptophyta, Cyanobacteria, Spirochaetes, Chloroflexi and unclassified sequences. Firmicutes was the predominant phyla in Snuff, but Cyanobacteria, Spirochaetes, and Streptophyta were also present in equal proportion.

**Figure 3:**
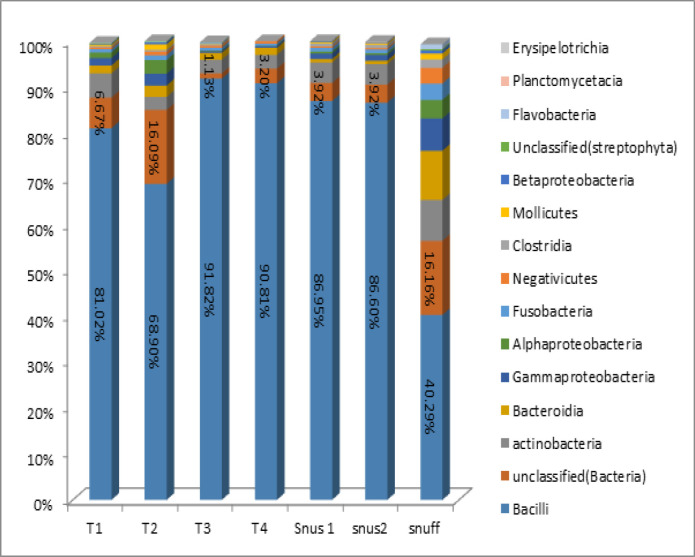
Bacteriome of Smokeless Tobacco Products – Class Level. (Stacked bars showing relative abundance of bacterial class identified in each of the ST product)

Class level bacteriome of each Tobacco sample is shown in Figure 3. A total of 36 classes were identified across all STPs (Graph representing only bacteria with relative abundance). Bacilli accounted for the predominant class in all the Tobacco products followed by Actinobacteria, Bacteroidia, Gamma proteobacteria, Alpha proteobacteria and the class which accounted for less than 1% include Fusobacteria, Negativicutes, Clostridia, Mollicutes, Betaproteobacteria and Flavobacteria. In T1 chewable Tobacco, Bacilli accounted for the predominant class in all the Tobacco products followed by Actinobacteria, Bacteroidia, Gamma proteobacteria, Alpha proteobacteria and the class which accounted for less than 1% include Fusobacteria, Negativicutes, Clostridia, Mollicutes, Betaproteobacteria and Flavobacteria. In T2 chewable Tobacco, Bacilli class was the highest and accounted more than 68% followed by Alphaproteobacteria, Actinobacteria, Gammaproteobacteria, Bacteroidia, Mollicutes fusobacterium and the class present less than 1% were Negativicutes, Betaproteobacteria, Clostridia and Flavobacteria. In T3 and T4 chewable Tobacco, Bacilli was the highest and accounted more than 90% followed by Actinobacteria, Bacteroidia and Fusobacteria, Negativicutes, Gammaproteobacteria, Betaproteobacteria and Mollicutes. In Snus 1 and Snus 2, Bacilli class was the highest and accounted more than 80% followed by Actinobacteria and Bacteroidia. In snuff, Bacilli class was the highest and accounted more than 35% followed by Bacteroidia, Actinobacteria, Gammaproteobacteria, Alphaproteobacteria, Fusobacteria, Negativicutes, Clostridia, Betaproteobacteria, Flavobacteria.

**Figure 4:**
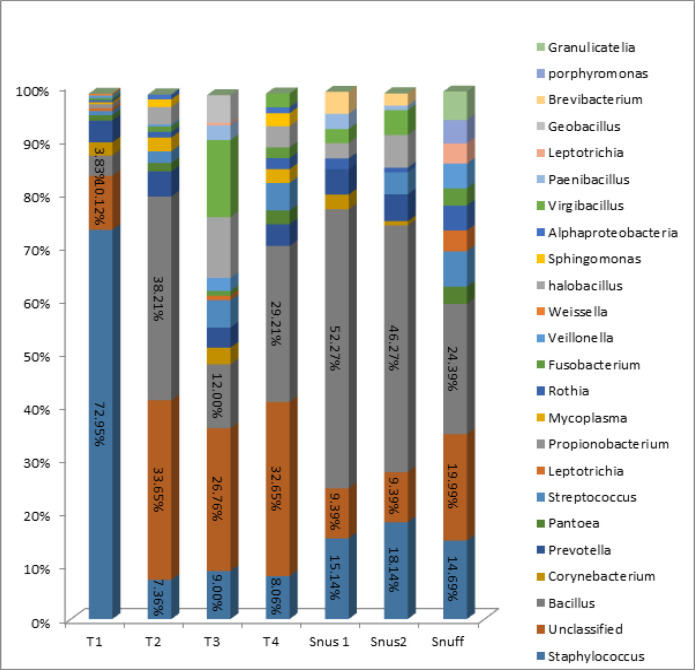
Bacteriome of Smokeless Tobacco Products – Genus Level. (Stacked bars showing relative abundance of bacterial genus identified in each of the ST product)

The genus-level bacteriome of each of the ST sample is presented in Figure 4. A total of 493 genera were identified, 24 of which were in relative abundance. The genus staphylococcus accounted for more than 75% of the reads in T1 chewable Tobacco followed by Bacillus and Corynebacterium and other genera less than 2% were prevotella, Pantoea, Streptococcus, Leptotricia, Propionibacterium, Rothia, Fusobacterium, Veillonella and Weisella. In T2 chewable Tobacco, >30% accounted for the unclassified types followed by Bacillus, Staphyloccocus, Prevotella, Virgibacillus, Halobacillus, and Streptococcus. In T3 and T4 chewable Tobacco, the genus bacillus was the highest accounting for more than 40% followed by staphylococcus, virgibacillus, halobacillus, unclassified derived from bacteria, Prevotella, Corynebacterium, Streptococcus. In both Snus 1 and snus 2, Bacillus accounted for more than 50% followed by Staphylococcus, Prevotella, Virgibacillus, Brevibacterium and the genus present in the range of 0-2% are Halobacillus, Oceanobacillus, Lactobacillus, Streptococcus, Bacteroides, Paenibacillus, Corynebacterium and Rothia. In Snuff, a total of 275 genres were identified. Out of which, Staphylococcus was the highest and accounted more than 15% followed by Bacillus, Prevotella, Streptococcus, Rothia, Veillonella, Fusobacterium and the genus present in the range of 0-2% are leptotrichia, Porphyromonas, Sphingomonas, Granulicatella, Pantoea and Actinomyces.

**Figure 5:**
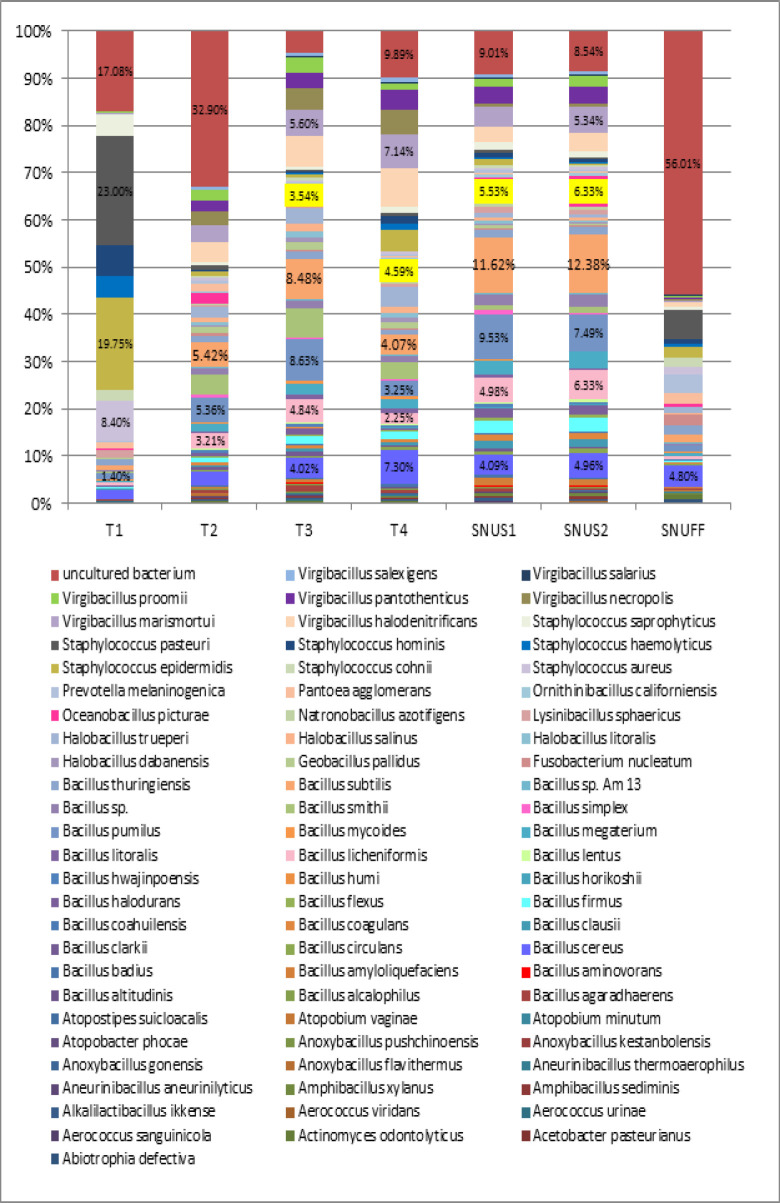
Bacteriome of Smokeless Tobacco Products – Species Level. (Stacked bars showing relative abundance of bacterial species identified in each of the ST product)

Figure 5 illustrates the species-level bacteriome of each of the ST product. A total of 2369 species were identified across all Tobacco products, out of which ≥1 relative abundance was seen in 76 species. The species number varied across samples with T3 Tobacco and snus showing the highest diversity of species. The most abundant species in T1 chewable Tobacco were Staphylococcus pasteuri followed by Staphylococcus epidermidis. In T2 chewable Tobacco products, the predominant species was Bacillus pumilus, Virgibacillus halodentrificans, Virgibacillus marismortoui, virgibacillus necropolis. In T4, the predominant species was Virgibacillus halodentrificans, Staphylococcus epidermidis, Oceanobacillus picturae and Bacillus subtilis. In snus 1 and snus 2, Bacillus pumilus and Bacillus subtilis followed by uncultured bacterium, Virgibacillus marismortoui, Oceanobacillus picturae. In snuff, the highest identified were uncultured bacterium, followed by Bacillus cereus, Bacillus pumilus and Fusobacterium nucleatum. 

## Discussion

Tobacco use may be defined as any habitual use of Tobacco plant leaf or its products, but the Tobacco product itself is classified based on its use. The predominant use of Tobacco is by smoke inhalation of cigarettes, pipes or cigars and the other way of using Tobacco is without burning called smokeless Tobacco products. These smokeless Tobacco refers to a variety of Tobacco products that are either chewable, snus and snuff (Popova and Ling, 2013)

Although there is enough evidence regarding how these STPs are responsible for causing cancer, some consumers of smokeless Tobacco products believe on the myth that “smokeless Tobacco products are less harmful”. Apart from being carcinogenic in nature, a potential risk that is linked with these products is that they could carry microorganisms which are pathogenic and might result in the evolution and progression of an infectious disease. This is a matter of solicitude because these products are held in close contact with oral mucosa for a longer duration because of its method of usage. Another matter of concern is that the contamination of these products with microbes can lead to the formation of toxic metabolites that may be harmful to the consumers (Naveen Kumar et al., 2016; Chopyk et al., 2017).

A group of chemical constituent found in smokeless Tobacco is Tobacco speciﬁc nitrosamines (TSNA), which is the most important Tobacco associated carcinogen. TSNA is a major risk factor for human health and members of this class of chemicals, 4-(methylnitrosamino)-1-(3-pyridyl)-1-butanone (NNK) and N-nitrosonornicotine (NNN) are listed as Group I carcinogens (carcinogenic to humans) by IARC. The critical nitrosating agent which is present in STPs is the nitrite nitrogen as the TSNA levels seem to correlate with nitrite in STPs (Bhisey, 2012). These nitrites are formed by the reduction of nitrate which is thought to be mediated by bacteria associated with the Tobacco products. The magnitude of these nitrosating reactions depends on complex interacting factors, but is generally thought to be steered by a combination of moisture content and temperature.

It has been shown that moisture content plays a role in NNN levels because STPs with higher water content supports microbial growth with subsequent accelerated microbial enzymatic reduction of nitrates to nitrite, which promotes nitrosation and resultant elevated NNN levels (Ammann et al., 2015). STPs may contain an array of hydrocarbons which may be a source of carbon to some bacterial communities and potentially provide them with a growth advantage over others in the Tobacco environment.

The different STPs are also blended with various additive assortments such as methanol by commercial manufacturers, thereby resulting in varying nitrate levels, resulting in different arrangements of microbial community with resultant difference in their carcinogenic potential (Stepanov et al., 2008). 

Collectively, the above findings suggest that the pH and moisture content plays a role in microbial growth in smokeless Tobacco products. Although, ample information is available regarding the differences in chemical composition, not much data exist on the bacterial populations present in STPs. Thus there was a strong need for filling the knowledge gaps related to microbial population present in STPs.

Previous studies on bacterial communities present in STPs have relied on conventional culture-based methods that could likely under-represent community diversity. Only a few studies have utilized culture-independent methods such as 16S rDNA to analyze Tobacco products. But one of the limitation of these studies is that, most of these methods have utilized only the standard methods of interrogating the V3-V4 region alone of the bacterial genome, which could potentially give only 60% of bacterial coverage. Whereas, a panel covering all V1-V9 regions provides sensitive detection and accurate representation of each species in the sample compared to standard methods interrogating the V3-V4 region alone. 

Hence, the primary objective of this study was to provide a preliminary and baseline data of the microbial populations that can be present in smokeless Tobacco products using 16sDNA analysis covering all hypervariable regions of the bacterial genome (V1-V9) and also to assess the possible relationship of pH and moisture content of these products on the bacterial communities present in the STPs. The present study included a total of 7 different commercially available smokeless Tobacco products (4 chewable Tobaccos, 2 snus, 1 snuff) purchased from a retail store from North India.

*pH levels in smokeless Tobacco products*

The study results showed the snus examined had the highest pH with alkaline values ranging from 8.15 and 8.50, chewable Tobacco and snuff had pH in the same range which had acidic values ranging from 5.63 to 6.09 and 5.62 respectively.

The results of the present study were in accordance with Andersen et al., (1993) in which three smokeless Tobacco products were evaluated and found chewable Tobacco and snuff had acidic pH of 6.1 and 6.7 respectively and concluded that there is a rise in a pH level on increase in the moisture content.

Richter and Spierto (2003) showed results in which most of the moist snuff products had alkaline pH than the loose leaf Tobacco products and concluded that pH of the Tobacco product is one of the determinant factor in deciding the rapid absorption of nictoine from the product.

Stepanov et al., (2008) showed results in which different Swedish brands of Snus varying geographical locations had varying pH ranging from 6.64-6.85 and another brand of snus (camel snus) had basic pH ranging from 7.23 – 8.23 suggesting that there is a regional variation in the physical characters of these products. 

Zakilluah et al., (2012) studied the potential toxicity of Naswar, a snus type Tobacco which was highly basic, averaging 8.56, and suggested an alkaline product favours the formation of Tobacco specific amines thus making the product potentially toxic. 

It is suggested that pH of a smokeless Tobacco product is a prime determinant in the quantity of unprotonated nicotine which affects the bioavailability of nicotine. Nicotine exists as protonated and unprotonated forms in the Tobacco product and the unprotonated form is the one which is rapidly absorbed from the mouth and like any other drug, the rate of absorption is a major factor for addiction. Hence most of the manufacturers add additives to alter the pH level of the Tobacco to increase the amount of unprotonated nicotine. Another factor which controls the nicotine absorption is the amount of moisture content. Manufacturers add additives to increase the salivation of the user such as acetic acid which will moisten the Tobacco held in the mouth and facilitate the extraction of nicotine (Richter and Spierto, 2003).

Prabhakar et al., (2013) showed results which were in accordance of our study that the Snus and snuff examined had alkaline pH value (ranging from 9.18 - 9.27) and suggested that pH of the Tobacco products is a determinant for faster absorption of nicotine.

Han et al., (2016) showed results in which there were differences in the pH levels among the different product types, with moist snuff having higher pH levels (7.4 to 8.2) and chewing Tobacco having the lowest pH levels (5.5 to 6.0) and suggested that pH and moisture content are directly proportional and the moist samples could support bacterial growth. 

Put together, the above observed findings suggest that pH values depend on the type of the Tobacco product and regional variations may occur. Also, this discrepancy in the pH values may be due to the fact that Tobacco naturally occurs in an acidic form and therefore is slow to release free-base nicotine unless buffered to alkaline levels. Hence, the difference in pH could be attributed to the varying manufacturing methods, additives used to alter alkalinity, and the moisture content in the product.

*Moisture content in smokeless Tobacco products*

The study results showed that snus products had the highest moisture content followed by chewable Tobacco. The moisture content of snus had values ranging between14.3% to 15.4%, chewable Tobacco had values ranging between 10.1% to 11.8% and snuff had a moisture content of 11.4%.

Prabhakar et al.,(2013) while evaluating the pH and moisture content of Tobacco products utilized two different methods (Karl Fischer titration versus gas chromatography) and suggested that there could be variations in the moisture content based on the method evaluated. However, on correlating the pH and moisture content, there was a direct relationship between these characters.

Ammann et al., (2016) showed results in which moisture levels of chewing Tobacco ranged from 7.2 to 21.3%, snus moisture levels ranging from 20.2% to 44.0% and snuff moisture levels ranged from 3.9% to 7.9%. Further they also compared their data set with previously reported data on the same products and found a variation in the moisture content of these products and suggested that use of different methods for estimation and composition changes of the product may be factors contributing to the observed differences in the moisture content.

Han et al., (2016) studied the correlation of moisture content to bacterial colonization of bacterial products and suggested that moist samples had the highest bacterial contamination, both in terms of number and diversity, which coincided with alkaline pH and high moisture levels in the product.

Put together, these findings suggest that the percentage of moisture levels in the Tobacco products might vary depending on the method employed, compositional changes and duration of storage of the product. However, there is a positive relationship between pH and moisture content. Moisture content plays an important role in converting the nicotine into its active absorbable form and also plays a major role in harboring more diversified and increased quantity of microbiome in the Tobacco product. 

*Relative abundance of microbial communities*

A total of 11 phyla were identified in the smokeless Tobacco products. Phylum Firmicutes accounted for ≥80% of sequences all Tobacco products except T7 (snuff). The other phyla were Actinobacteria, Proteobacteria, Bacteroidetes, Fusobacteria, Tenericutes, Streptophyta, Cyanobacteria, Spirochaetes, Chloroflexi and unclassified sequences. T7 Snuff had Firmicutes as the predominant but Cyanobacteria, Spirochaetes, and Streptophyta were also present in equal proportions. 

A total of 493 genera were identified, 24 of which were in relative abundance. The genus Staphylococcus accounted for more than 75% of the reads in T1 chewable Tobacco followed by Bacillus and Corynebacterium and other genera less than 2% were prevotella, Pantoea, Streptococcus, Leptotricia, Propionibacterium, Rothia, Fusobacterium, Veillonella and Weisella. In T2 chewable Tobacco, >30% accounted for the unclassified types followed by Bacillus, Staphyloccocus, Prevotella, Virgibacillus, Halobacillus, and Streptococcus. In T3 and T4 chewable Tobacco, the genus Bacillus was the highest accounting for more than 40% followed by staphylococcus, Virgibacillus, Halobacillus, Prevotella, Corynebacterium, and Streptococcus. The genus Bacillus accounted for the majority of the genera in both snus and snuff Tobacco product. In T7 snuff Granulicatella, Paenibacillus and Virgibacillus were present.

Bacillus accounted for the predominant species in majority of the reported literature and it is due the ability of the organisms to form endospores, which are capable of resisting dryness, extreme temperatures and endogenous factors that could inhibit the growth and reproduction of vegetative organisms.

Although, majority of the Bacillus species that were detected are common and usually not associated with acute illnesses; Bacillus licheniformis is a potential cause for pulmonary inflammation and Bacillus cereus causes food poisoning by production of emetic toxin and regarded as one of the important causes of food poisoning in India (Rubinstein and Pederson, 2002; Anita and Swaid, 2015).

Another potential concern is that some species of Bacilli including Bacillus subtilis and licheniformis, have the ability to reduce nitrates, which are important precursors for nitrosation of nicotine to form TSNAs, which are carcinogenic. The presence of these organisms in the STPs suggests the probability of TSNA formation after packaging of the product. This phenomenon is compatible with results of previous studies that found the levels of TSNAs are higher in the presence of these Bacillus in certain STPs and the levels of TSNAs increased over time when stored under ambient conditions (Djordjevic et al., 1993; Fisher et al., 2012).

Among Staphylococcus, strains of S. epidermidis and S. hominis and S. pasteuri were the predominant species present. Not only S. epidermidis and S. hominis are potential nitrate reducers, they also present with possible health concerns as opportunistic organisms, especially for individuals with compromised immune status. Both these staphylococcal species have been reported to cause bacterial endocarditis, due to transmission of these pathogens to the heart through the bloodstream. A combination of poor oral hygiene with the presence of periodontal pathogens in the STP may result in individuals having inflammation of the gingiva, which theoretically may support the entry of bacteria.

Staphylococcus pasteuri is a Gram positive, coagulase-negative organism, which is emerging as a causative agent for nosocomial infections and a blood derivatives contaminant. This bacterium has recently appeared to express resistance against several classes of antibiotic compounds, such as methicillin, macrolides, tetracyclines, chloramphenicol, and streptomycin (Savini et al., 2009). 

Oceanobacillus picturae is another bacillus which was found in abundance in the chewable Tobacco products and some species are accompanied with fermentation of foods and hence it is logical to assume that these organisms might be present in the Tobacco products. Interestingly, some of the Oceanobacillus have been identified as being more alkaliphilic and yet the chewing Tobacco samples had acidic pH (Yumoto et al., 2005).

Several genera identified from the STPs in the study have been shown to be part of the habitat of the oral microbiome. The STPs were positive for periodontal pathogens from the genera Eubacterium, Porphyromonas and Prevotella, although they were relatively low in numbers and present only in few samples. Other genera identified included Lactobacillus and Actinomyces, which have established roles in the pathogenesis of dental caries.

Also, there were few novel bacteria which were identified such as Virgibacillus pantothenticus, which is a Gram-positive, spore-forming, aerobic, mesophilic, and halotolerant bacterium and has been reported to be a clinical opportunistic pathogen causing liver abscess and sepsis (Jie-ping et al., 2015). 

In summary, the pH and moisture levels detected in the STP samples analyzed in the study are compatible with data reported in previous literature. The snus samples had the highest levels of bacterial communities in terms of diversity, which may be related to their high moisture levels. Some of the chewing Tobacco (T1) and snus samples harbored significantly fewer bacteria than the snuff samples, indicating that the relatively high levels of bacteria in moist snuff warrants greater scrutiny in future studies. 

The present study findings provide baseline data on the microbial content of STPs, which is clinically important to assess its potential risk to the users, aid in stringent manufacturing processes and assist health care administrators in establishing regulations. The fact that many of the identified bacterial species possess the ability to reduce nitrate to nitrite and thus potentially contributing to the production of TSNAs, which are highly carcinogenic also necessitates further attention in future studies.

**Table 1:** Moisture Content and pH of the Tobacco Samples

S. No	Sample ID	Type	pH	Moisture Content
1	T1_CW	Chewable tobacco	5.63	10.10%
2	T2_CW	Chewable tobacco	5.85	11%
3	T3_CW	Chewable tobacco	6.09	11.80%
4	T4_CW	Chewable tobacco	5.95	11.10%
5	T5_Snus	Snus	8.5	15.40%
6	T6_Snus	Snus	8.15	14.30%
7	T7_Snuff	Snuff	5.62	11.40%

**Table 2:** Alpha Diversity

S. No	Sample ID	Type	α-diversity
1	T1_CW	Chewable tobacco	33 species
2	T2_CW	Chewable tobacco	118 species
3	T3_CW	Chewable tobacco	152 species
4	T4_CW	Chewable tobacco	136 species
5	T5_Snus	Snus	168 species
6	T6_Snus	Snus	142 species
7	T7_Snuff	Snuff	99 species

Alpha diversity is the measure of diversity in a single sample. T5_Snus shows high number of significant species in the sample and T1_CW (chewable tobacco) with the lowest number of species diversity.
